# The evolution of robotic-assisted thoracic surgery: current platforms, emerging technologies, and future perspectives - a narrative review

**DOI:** 10.1007/s11701-026-03722-w

**Published:** 2026-08-06

**Authors:** Khrystyna Kuzmych, Filippo Lococo, Dania Nachira, Alessia Senatore, Giuseppe Calabrese, Maria Letizia Vita, Leonardo Petracca-Ciavarella, Maria Teresa Congedo, Elisa Meacci, Stefano Margaritora

**Affiliations:** Department of General Thoracic Surgery, Fondazione Policlinico Universitario “A. Gemelli”, IRCCS, Università Cattolica del Sacro Cuore, Rome, 00168 Italy

**Keywords:** Robotic-assisted thoracic surgery, RATS, Thoracic surgery, Robotic platforms, Artificial intelligence, Minimally invasive surgery

## Abstract

Robotic-assisted thoracic surgery (RATS) has undergone rapid technological evolution over the past two decades, progressively expanding from early minimally invasive applications to increasingly complex thoracic procedures. This narrative review examines the historical development of robotic platforms in thoracic surgery, the emergence of new approaches and alternative robotic systems, and future technological perspectives. A literature search was conducted using PubMed/MEDLINE, Embase, Scopus, Web of Science Core Collection, and the Cochrane Library to identify relevant English-language publications through May 2026. Early robotic systems established the feasibility of robotic thoracic procedures, particularly mediastinal surgery and selected pulmonary resections, while also defining the technical limitations that shaped early practice, including demanding docking, arm-collision issues, dependence on bedside assistance, and lack of console-controlled stapling. The da Vinci Xi refined multiport robotic surgery through improved arm design, overhead boom architecture, simplified docking, and integrated stapling, supporting greater standardization and broader adoption of robotic thoracic surgery. In parallel, reduced-port approaches, including biportal and uniportal RATS, emerged through technical adaptation of existing platforms. The da Vinci SP introduced a dedicated single-port architecture, whereas the da Vinci 5 represents a further step toward haptic feedback and data-driven robotic surgery. Emerging systems such as Versius, Hugo RAS, Toumai, and Shurui SP reflect a diversifying robotic landscape, with modular, portable, and lower-cost design, that may influence future access and adoption. Future developments include artificial intelligence, augmented reality, digital twins, autonomous assistance, and remote surgery. Overall, robotic thoracic surgery is evolving from a purely mechanical platform toward a more integrated digital surgical environment, although the clinical impact, accessibility, training requirements, and long-term benefits of many emerging technologies remain to be fully established.

**Supplementary Information:**

The online version contains supplementary material available at 10.1007/s11701-026-03722-w.

## Introduction

The surgical management of thoracic pathologies has undergone a profound transformation over the past three decades. The transition from open thoracotomy to video-assisted thoracoscopic surgery (VATS) in the 1990s established the principle that minimally invasive approaches could achieve equivalent oncologic outcomes while reducing postoperative morbidity, pain, and length of stay [1]. However, VATS remained technically challenging because of two-dimensional visualization, limited depth perception, and restricted instrument articulation, with a relatively long learning curve [2].

**Fig. 1:**
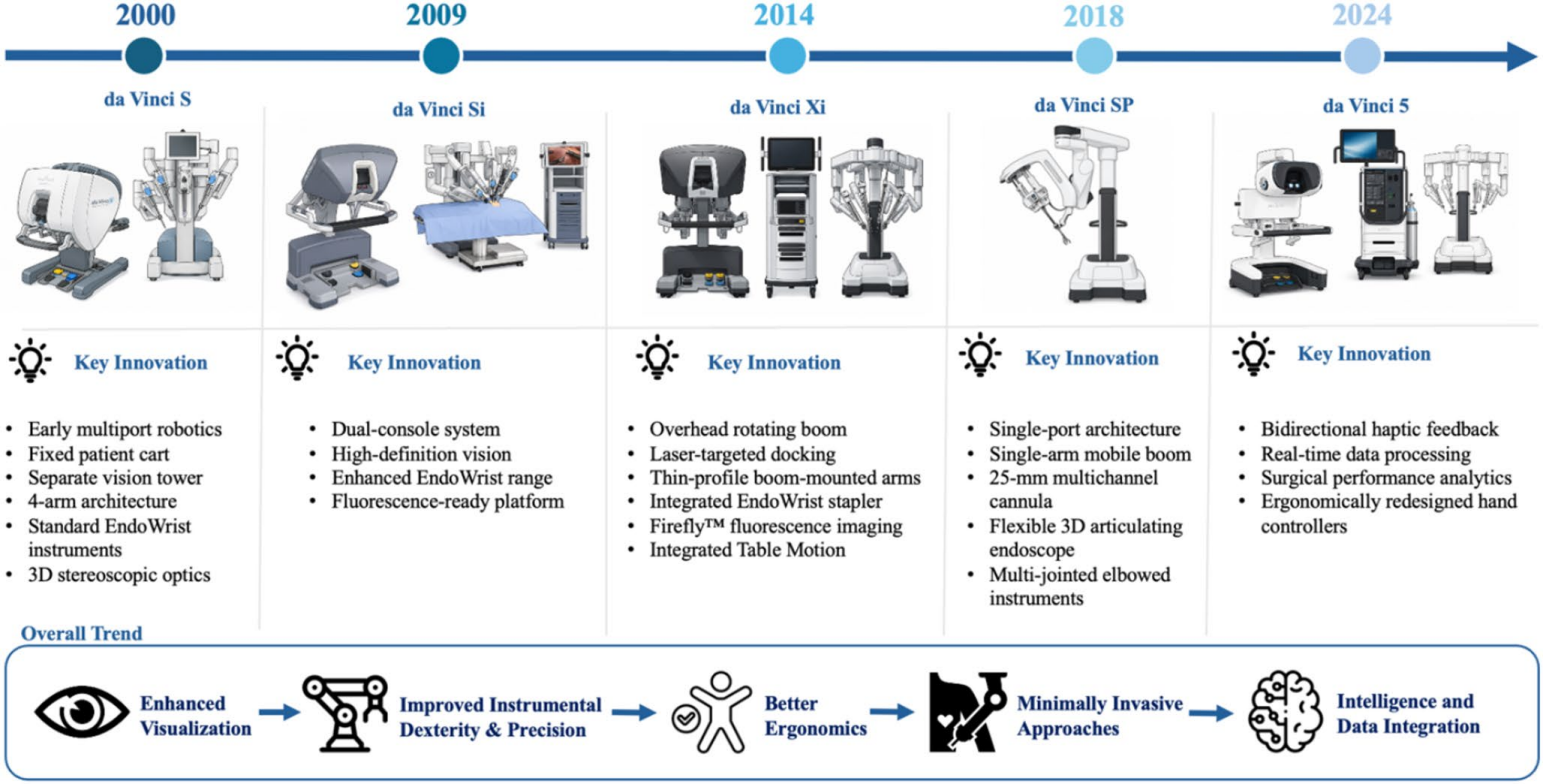
Evolution of da Vinci robotic surgical platforms and major technological innovations

Robotic-assisted thoracic surgery (RATS) emerged as a technological evolution designed to overcome these constraints (Fig. 1). The robotic platform offered several advantages, such as three-dimensional, high-definition visualization with magnification, wristed instruments capable of mimicking the movements of the human hand with seven degrees of freedom, tremor filtration, and improved surgeon ergonomics [1, 3]. Observational studies have suggested that these features may potentially improve operative performance, including more precise tissue dissection, reduced intraoperative blood loss, lower conversion rates to thoracotomy, and enhanced lymphadenectomy with a greater number of nodal stations sampled [4–6].

The rapid evolution of robotic platforms over the last two decades has fundamentally shifted from the robot as a passive surgical instrument to an integrated surgical ecosystem. The first-generation da Vinci system, introduced in 2000, was a multiport platform with limited stapling capability and cumbersome docking [3]. Successive iterations addressed these limitations, and the introduction of advanced systems around 2014 accelerated worldwide adoption of robotic thoracic surgery. The landmark multicenter randomised controlled RVlob trial demonstrated oncological non-inferiority of RATS compared with VATS, with 3-year overall survival of 94.6% versus 91.5%, respectively, although definitive evidence remains limited [7]. At the same time, the field is now entering a new phase driven by multiple innovations. Single-port robotic platforms have demonstrated feasibility and safety across a range of thoracic procedures [8]. The da Vinci 5 system, approved in 2024, introduced the first integrated force feedback haptic technology, enabling surgeons to sense push and pull forces, addressing what has long been considered the most significant limitation of robotic surgery [9, 10]. Meanwhile, artificial intelligence is being integrated across the surgical pathway, from preoperative planning with nodule detection and risk stratification, to intraoperative navigation using augmented reality and real-time decision-support systems, to postoperative predictive analytics for complication detection [11, 12].

The competitive landscape has also shifted. The emergence of alternative platforms, including the Versius system (CMR Surgical), Shurui SP (Beijing Shurui Technology Co), and others, has introduced modular architectures, open-console designs, smaller instruments, and the potential for reduced costs [13–15].

Yet as the technology accelerates, fundamental questions remain unresolved. Does the evolution of robotic platforms translate into measurably improved patient outcomes, or does it primarily enhance surgeon experience and ergonomics? The impact of technological evolution on the learning curve is similarly uncertain. Data suggest that the steepest improvements in console time occur within the first 20–40 cases for anatomical lung resection, with continued efficiency gains beyond 100 cases, but whether next-generation features such as haptic feedback and AI-assisted navigation will shorten or paradoxically prolong the learning curve by introducing new layers of complexity is unknown [16].

This narrative review examines the current state and future trajectory of robotic-assisted thoracic surgery, with particular attention to the clinical evidence supporting emerging technologies, the evolving training paradigm, and the critical question of whether the next generation of robotic platforms will fulfill their promise of improved outcomes.

## Materials and methods

This narrative review provides an overview of the evolution of robotic thoracic surgery, including current robotic platforms, emerging technologies, surgical applications, training paradigms, and future perspectives.

A literature search was conducted across PubMed/MEDLINE, Embase, Scopus, Web of Science Core Collection, and the Cochrane Library to identify relevant English-language publications available through May 2026. The search was designed to identify relevant evidence on robotic thoracic surgery, including current platforms, emerging technologies, clinical applications, surgical training, and future developments. Studies were selected purposively according to their relevance and scientific contribution to the topics addressed. To enhance transparency and reproducibility, the complete search strategy, eligibility criteria, study selection process, and PRISMA-like flow diagram are provided in the Supplementary Material. Given the broad scope of the topic and the heterogeneity of the available evidence, findings were synthesized narratively and organized into thematic sections.

There was no patient or public involvement in the design or conduct of this review.

## Historical evolution of robotic platforms in thoracic surgery

### Early robotic systems: the da vinci standard platform

The introduction of robotic surgery into thoracic practice began shortly after FDA approval of the da Vinci system in 2000. Among the earliest reported experiences, Bodner and colleagues described a small single-center feasibility series of thymectomies, esophageal dissections, and a single lobectomy performed with the original da Vinci platform in 2001–2003, concluding that the rigid anatomy of the chest was an ideal condition for robotic surgery but noting that a major limitation is the lack of more appropriate instruments, especially during pulmonary lobectomy [17]. These early systems, the da Vinci Standard, S, and Si, offered transformative advantages in visualization and dexterity over conventional thoracoscopic instruments, including three-dimensional high-definition optics, articulating instruments with seven degrees of freedom, and tremor filtration Table 1. However, the platforms had some technical constrains. The absence of integrated robotic stapling required a trained bedside assistant to place, manipulate, and fire the stapler around major vascular structures, thereby introducing procedural variability and potential risk [3, 17, 18]. The bulky robotic arms of these early systems were prone to external collisions, while their fixed positions relative to the base constrained multi-quadrant access [19, 20]. In addition, instrument availability was limited, and the cumbersome docking process demanded meticulous port placement with minimal margin for error [21, 22].

Despite these limitations, thoracic surgery adopted robotics with notable enthusiasm, particularly for procedures in which the platform’s advantages were most evident. Mediastinal surgery, especially thymectomy, emerged as the paradigmatic application. The narrow, deep anterior mediastinum, where precise dissection around the phrenic nerves and great vessels is paramount, proved ideally suited to the robot’s magnified 3D-view and articulating instruments [23, 24]. In single-center retrospective series (*n* = 106), Rückert and colleagues reported consecutive robotic thymectomies with zero mortality and a 2% morbidity rate, noting that the da Vinci system allowed technical refinements of thoracoscopic thymectomy with a shorter learning curve [23]. By 2014, robotic thymectomy had grown from 6% to 14% of all thymectomies nationally in the United States, reflecting increasing adoption based on largely accumulating observational experience [25].

Robotic lobectomy evolved gradually during the Si era, with multiple techniques varying in the number of robotic arms (three versus four), the use of CO₂ insufflation, the timing and location of the utility incision, as well as port positioning [26]. These differences reflected the surgeon’s need to adapt to the platform’s limitations rather than a standardized approach. The Si platform fundamentally shaped the early robotic thoracic surgery principles, including meticulous geometric port planning to minimize arm collisions, a central operative role for the bedside assistant, functioning as an extension of the console surgeon for stapling, retraction, and lung manipulation, and the console-based workflow requiring new models of surgeon–team communication, as the operating surgeon was physically separated from the patient and had no view of bedside activity [26]. Notably, comparative data from this era are limited to retrospective series and registry analyses; no randomised trials were conducted comparing early RATS with VATS or open thoracotomy for lobectomy.

Early training programs also reflected these challenges. The learning curve for robotic thymectomy was steep, with operative time plateauing after 15–20 cases, while anatomic lung resection required 20–40 cases for initial proficiency [16]. Paradoxically, although the robotic platform offered superior dexterity and more intuitive movements compared with VATS, the overall learning curve was comparable to or even slightly longer than the VATS when measured by surgical failure rates rather than operative time alone [27]. This finding illustrates an important distinction: improved instrument capability does not automatically translate into faster clinical proficiency. Trainees had to adapt to the absence of haptic feedback, rely exclusively on visual cues, master remote console communication, understand robotic arm mechanisms and collision geometry, and gain proficiency in bedside assistance before console operating [28]. This paradox remained a recurring theme as newer platforms introduced additional technological innovations.

### The da vinci Xi era: standardization and expansion

The introduction of the da Vinci Xi in 2014 represented a radical change both in form and functionality, accelerating the exponential growth of robotic thoracic surgery [19]. The Xi’s fourth-generation architecture addressed several technical limitations through thinner and longer arms with reduced external profiles that decreased arm collisions, an overhead boom enabling multi-quadrant and multi-cavity access without repositioning the patient cart, laser-guided docking, and the integration of a robotic vascular stapler controlled directly by the console surgeon [18–20]. This innovation transformed robotic lobectomy by enabling port configuration such as the “five on a dice” setup, allowing the console surgeon to perform a near-solo procedure with minimal bedside assistance [18]. In addition, the improved mobility of the Xi boom made port placement less restrictive, compensating for suboptimal port geometry and decreasing arm collisions [19]. These changes collectively transformed robotic lobectomy from a technically demanding procedure into a more reproducible and standardized operation.

The Xi era produced the first randomized controlled trials comparing RATS with VATS. The landmark multicenter RVlob trial (*n* = 394) demonstrated oncological non-inferiority of RATS, with a 3-year overall survival of 94.6% versus 91.5% for VATS but did not establish superiority [7]. The multicenter ROMAN trial (*n* = 83) found no significant differences in perioperative outcomes between RATS and VATS, although robotic surgery achieved a more extensive lymph node dissection [29]. The ongoing international RAVAL trial is evaluating patient-reported health utility, cost-effectiveness, and long-term oncological outcomes, with preliminary reports suggesting comparable or modestly improved health-related quality of life following robotic surgery; however, definitive peer-reviewed results are still awaited [30]. Similarly, a single-center Italian prospective randomized trial by Catelli et al. (*n* = 75) reported largely comparable perioperative outcomes between RATS and VATS, with some differences in postoperative pain and pleural effusion favoring the robotic approach [31]. Collectively, the available randomized evidence indicates that RATS achieves perioperative and oncological outcomes comparable to those of VATS, although robust evidence demonstrating clear clinical superiority of the robotic approach remains lacking.

Observational data from this era also suggested additional benefits, In a single-center retrospective cohort comparison, Soliman and colleagues reported significantly fewer postoperative complications (15.2% in the mature robot period versus 39.1% in the pre-robot VATS era), shorter median length of stay (2 versus 4 days), and lower readmission rates (1.9% versus 11.8%), with multivariate analysis confirming the robot as an independent predictor of reduced complications [32].

The Xi era also expanded the indications for robotic thoracic surgery. In retrospective series from high-volume centers, robotic sleeve lobectomy became increasingly feasible and reproducible, with reported lower conversion rates (6.4% versus 18.2% for VATS) and greater lymph node yield [33]. Similarly, complex segmentectomies, including atypical and multi-subsegmental resections, were increasingly performed robotically without increased morbidity [34]. In esophageal surgery, the RAMIE trial, a multicenter randomized controlled trial, showed that robot-assisted esophagectomy achieved non-inferior, and potentially superior, 5-year overall survival compared with thoracoscopic esophagectomy, with improved upper mediastinal lymph node dissection and a shorter learning curve (approximately 35 versus 69 cases for proficiency) [35].

Although the Xi was designed as a multiport system, its versatility also enabled the development of reduced-port robotic approaches. Gonzalez-Rivas et al. [36, 37] recognized that the Xi’s arm geometry allowed progressive reduction from four incisions to two, and ultimately to one. In 2021, they performed the first fully robotic anatomic resection through a single intercostal incision. All robotic instruments, camera, and staplers pass through a single intercostal space, without rib spreading, and the surgeon operates with a direct-view analogous to uniportal VATS, while maintaining the advantages of a robotic system [36, 37]. In a multicenter retrospective cohort study (*n* = 106; 53 U-RATS vs. 53 multiport RATS) across nine European centers, uniportal RATS (U-RATS) and multiport RATS had equivalent R0 resection rates (100% in both groups) and comparable lymph node yields, while the U-RATS group showed shorter operative times (median 136 vs. 150 min), shorter postoperative length of stay (4 vs. 5 days), and no conversions to thoracotomy [38]. Progressively, U-RATS also extended to more technically demanding thoracic procedures. In the largest single-center retrospective series of U-RATS sleeve lobectomies to date (*n* = 134 consecutive cases using the da Vinci Xi), Wang and Gonzalez-Rivas [39] reported a 97.8% R0 resection rate and a median operative time of 185 min, including cases performed after induction therapy, where obliterated tissue planes and distorted vascular anatomy make surgical dissection particularly challenging.

A key reported safety advantage of U-RATS is the ability to rapidly undock and convert to either Uniportal VATS (U-VATS) or thoracotomy in case of intraoperative emergency [36, 37], unlike multiport RATS, which requires removing multiple trocars and repositioning the patient.

U-RATS remains technically demanding and appears most appropriate for surgeons with substantial prior experience in uniportal VATS and/or multiport RATS. Early learning curve studies suggest that adoption is feasible. Mercadante et al. reported a significant reduction in operative time across their initial 24 U-RATS lobectomies (single-center feasibility series) [40]. Similarly, Kaneda et al. confirmed feasibility and safety in an initial 25-case single-center experience, although operative time remained longer than U-VATS [41]. For this reason, Gonzalez-Rivas and colleagues have recommended a gradual transition pathway from multiport RATS to biportal RATS and finally to U-RATS [37, 38] to ensure complete spatial awareness and instrument coordination.

However, several important limitations should be acknowledged when interpreting the U-RATS evidence. The available evidence is derived predominantly from retrospective, non-randomized studies conducted predominantly by the technique’s pioneers or early adopters at high-volume centers, and no randomised controlled trials comparing U-RATS with multiportal RATS or U-VATS have been reported. Furthermore, long-term oncological outcomes, including disease-free and overall survival, remain unavailable.

Within this transition, biportal RATS (Bi-RATS) has emerged as an intermediate strategy, using only two intercostal incisions: a utility incision that accommodates the robotic camera, working instruments, specimen retrieval, as well as assistant instrumentation, if necessary, and a second accessory port dedicated to robotic stapling and additional instruments. This configuration creates a hybrid operative geometry that combines elements of uniportal in-line instrumentation with partial robotic triangulation, aiming to reduce chest wall access trauma while preserving the ergonomic and technical advantages of a fully robotic approach. In the first single-center retrospective comparative study of Bi-RATS and multiportal RATS lobectomies, Gu et al. found no significant differences in operative time, blood loss, lymph node yield, or complications, supporting the safety and feasibility of this approach [42]. Similarly, Watanabe et al. described a multi-institutional Japanese retrospective experience of dual-portal RATS (DRATS), characterized by low blood loss, no conversions, and no major complications, and proposed it as a useful preliminary step toward U-RATS [43], reinforcing the graduated pathways concept. In a single-center propensity score-matched comparison of Bi-RATS and U-VATS for anatomical lung resection, Nachira et al. [44] reported comparable perioperative outcomes, complication rates, and 6-month quality of life (EQ-VAS 78.1 vs. 78.9, *p* = 0.832), while showing a trend toward higher lymph node yield, particularly in segmentectomies, and significantly improved postoperative mobility (*p* = 0.045) in Bi-RATS. Operative time was longer with Bi-RATS (221 vs. 119 min), reflecting the early learning curve and the initially longer setup and docking times during the earliest cases, which decreased substantially with accumulating experience [44].

As with U-RATS, the Bi-RATS literature shares important limitations as all studies are retrospective and non-randomized, with relatively small sample sizes and no long-term oncologic follow-up. Bi-RATS may represent a complementary approach that adds robotic precision to a reduced-port philosophy, but whether it offers meaningful clinical advantages over established U-VATS or multiport RATS techniques awaits higher-level comparative evidence. Further multicenter studies are needed to define the role of these reduced-port approaches in routine clinical practice.

Overall, the Xi era had a dual effect on robotic thoracic surgery. On one hand, it standardized multiport robotic procedures by simplifying docking, improving arm maneuverability, reducing dependence on bedside assistance, and enabling console-controlled stapling. On the other hand, it stimulated further technical innovation, including biportal and uniportal robotic approaches that pushed the field toward increasingly less invasive strategies. Thus, the Xi platform simultaneously lowered the barriers to adoption and expanded the complexity of procedures performed robotically, creating the foundation for the subsequent evolution toward single-port platforms, modular robotic systems, and ultra-minimally invasive thoracic surgery.

### The da vinci SP platform: single-port surgery and new challenges

The da Vinci Single-Port (SP) system introduced a different architectural concept based on a flexible endoscope and three multi-jointed instruments deployed through a single 25-mm cannula, each capable of independent articulation. Unlike the Xi’s multiport design, the SP was engineered primarily for extracostal approaches such as subcostal or subxiphoid access [45], completely avoiding intercostal port placement, thereby reducing chest wall trauma, intercostal nerve injury, postoperative pain, and improving cosmetic outcomes. In thoracic surgery, the SP platform found its most natural application in mediastinal surgery via the subxiphoid approach, by enabling bilateral visualization of the mediastinum through a single small incision. Despite being primarily designed for extracostal access, surgeon in both Asia and Europe have also begun using the da Vinci SP via an intercostal approach by working with the external ball joint deflated to accommodate the intercostal space.

The clinical evidence for SP thoracic surgery remains at an early stage, consisting entirely of single-center, non-randomized series with small sample sizes. In a single-center, single-arm feasibility series (*n* = 23), Ito et al. reported consecutive SP mediastinal tumor resections via subxiphoid or subcostal approaches with no conversions, no severe complications, and R0 resection in all cases [46]. In another early single-center feasibility series (*n* = 17), Park et al. reported the first thoracic SP cases in 2022, using subxiphoid, subcostal, and intercostal approaches with a median operative time of 120 min, median pain scores of 3, and shorter chest tube duration compared with earlier da Vinci single-site surgery [45]. More recently, in a single-center retrospective comparative study (*n* = 60; 30 SP vs. 30 Xi), Kawaguchi et al. reported comparable perioperative outcomes between subcostal da Vinci SP and da Vinci Xi resections for stage I–III lung cancer, while observing fewer postoperative complications in the SP group despite occasional need for an additional access port [47]. Although preliminary, these early-adopter results support the feasibility of SP thoracic surgery while underscoring the current well-documented technical limitations of the platform.

A unique challenge is the intra-cannula instrument conflict as their elbow and wrist joints frequently operate beyond the camera’s field of view, increasing the risk of internal collisions and “skipping events”, which may cause unintended interactions with surrounding tissues [48, 49]. Consequently, surgeons must develop familiarity with the navigator view and the spatial relationships of off-screen instrument segments to anticipate and prevent these collisions, especially during the early learning curve. Camera control also differs substantially from conventional multiport systems, requiring adaptation to the flexible endoscope’s dual joggle joints. In addition, because SP currently lacks a dedicated robotic stapler, stapling must be performed by the bedside assistant using conventional staplers. This requires careful coordination to avoid conflicts with the camera and working instruments, despite the inherently limited bedside access [50]. Moreover, several series reported the occasional need for supplementary ports in cases involving adhesions, high BMI, or complex anatomy, partially reducing the cosmetic and trauma-reduction advantages [47, 51]. All these technical challenges have profound implications for the learning curve. Although the SP system partially restores instrument triangulation though its articulating, elbowed instruments and flexible endoscope, it demands heightened awareness within a narrower field of view. These observations suggest a recurring theme in robotic platform evolution: newer platforms may expand technological advantages while simultaneously prolonging or reshaping the learning curve, requiring dedicated training that cannot be transferred from multiport robotic proficiency.

### The da vinci 5 platform and the introduction of haptic feedback

The da Vinci 5 (DV5), introduced in March 2024, represents the most important technological advance, primarily through the integration of force-feedback haptic technology, the first FDA-approved system to provide a sense of touch [8]. Surgeons can perceive push and pull forces, tissue tension, and pressure during dissection, retraction, and suturing, addressing one of the major limitations of robotic surgery over the past two decades. The absence of haptic feedback has had clear technical and educational consequences. A meta-analysis of 56 studies comparing robotic surgery with and without haptic feedback demonstrated that haptic sensation reduced average and peak applied forces, improved task accuracy and success rates [10]. The greatest benefits were observed among less experienced surgeons as haptic-enhanced simulations have been shown to reduce trainee workload, improve perceived performance, and accelerate progression to proficiency, particularly for complex tasks [52–54].

Several potential applications of the DV5 platform remain speculative. Haptic feedback may enhance dual-console mentoring by allowing trainees to directly perceive appropriate tissue-handling forces demonstrated by expert surgeons, although this concept remains largely unexplored. AI-based computer vision systems can identify surgical actions, recognize instruments and anatomy, and correlate performance directly from raw operative videos [55]. The real-time integration of these data creates the foundation for AI-assisted surgical assessment, including automated detection of suboptimal tissue handling, identification of technical errors, and longitudinal performance tracking [56]. Together, haptic feedback, digital analytics, and AI-assisted assessment make the DV5 not only an improved robotic platform, but the basis of an integrated training ecosystem. Objective performance data may increasingly guide curriculum development, competency assessment, and credentialing, shifting surgical education from subjective observation to an automated, data-driven evaluation. This transition represents a broader paradigm shift in surgical training and leads into future models of robotic education and skill acquisition. However, these applications remain conceptual; no clinical validation has been published to date. Whether these capabilities will shorten the learning curve or introduce new layers of complexity requiring additional training is unknown.

Taken together, the evolution of the da Vinci platforms have consistently improved platform functionality, surgeon ergonomics, and operative workflow through advances such as simplified docking, integrated stapling, improved arm architecture, single-port access, and haptic feedback. By contrast, evidence that these engineering innovations have translated into measurable improvements in patient outcomes remains less robust. Although observational studies suggest potential perioperative advantages, randomized evidence have largely demonstrated oncological non-inferiority and comparable perioperative outcomes relative to established thoracoscopic approaches. Thus, while technological evolution has undoubtedly enhanced the surgical experience and expanded technical capabilities, its incremental impact on patient outcomes remains an important area for future investigation.

**Table 1:** Evolution and main characteristics of da vinci robotic platforms in thoracic surgery

Platform	Year of introduction	System Design	Key Features	Single-Port	Haptic Feedback	Main Thoracic Surgery Impact
**da Vinci Standard / S**	Early 2000s	Multiport robotic platform with fixed patient cart	3D visualization, wristed instruments, tremor filtration	No	No	Enabled initial robotic thoracic procedures
**da Vinci Si**	2009	Multiport platform	Dual-console, enhanced visualization, greater instrument range	No	No	Expanded robotic lobectomy and training adoption
**da Vinci Xi**	2014	Overhead boom multiport platform	Laser-guided docking; integrated robotic stapler; thinner boom-mounted arms	No	No	Standardized workflow and reproducibility; accelerated global adoption.
**da Vinci SP**	2018	Single-port flexible platform	Single 25-mm cannula, flexible camera, intracorporeal articulation	Yes (subxiphoid/ subcostal)	No	Expanded reduced-port and extracostal approaches
**da Vinci 5 (DV5)**	2024	Multiport robotic platform	Bidirectional haptic feedback Integrated performance analytics Real-time system data processing	No	Yes	Introduced haptics and data-driven surgical assessment

## Alternative robotic platforms and market expansion

**Fig. 2:**
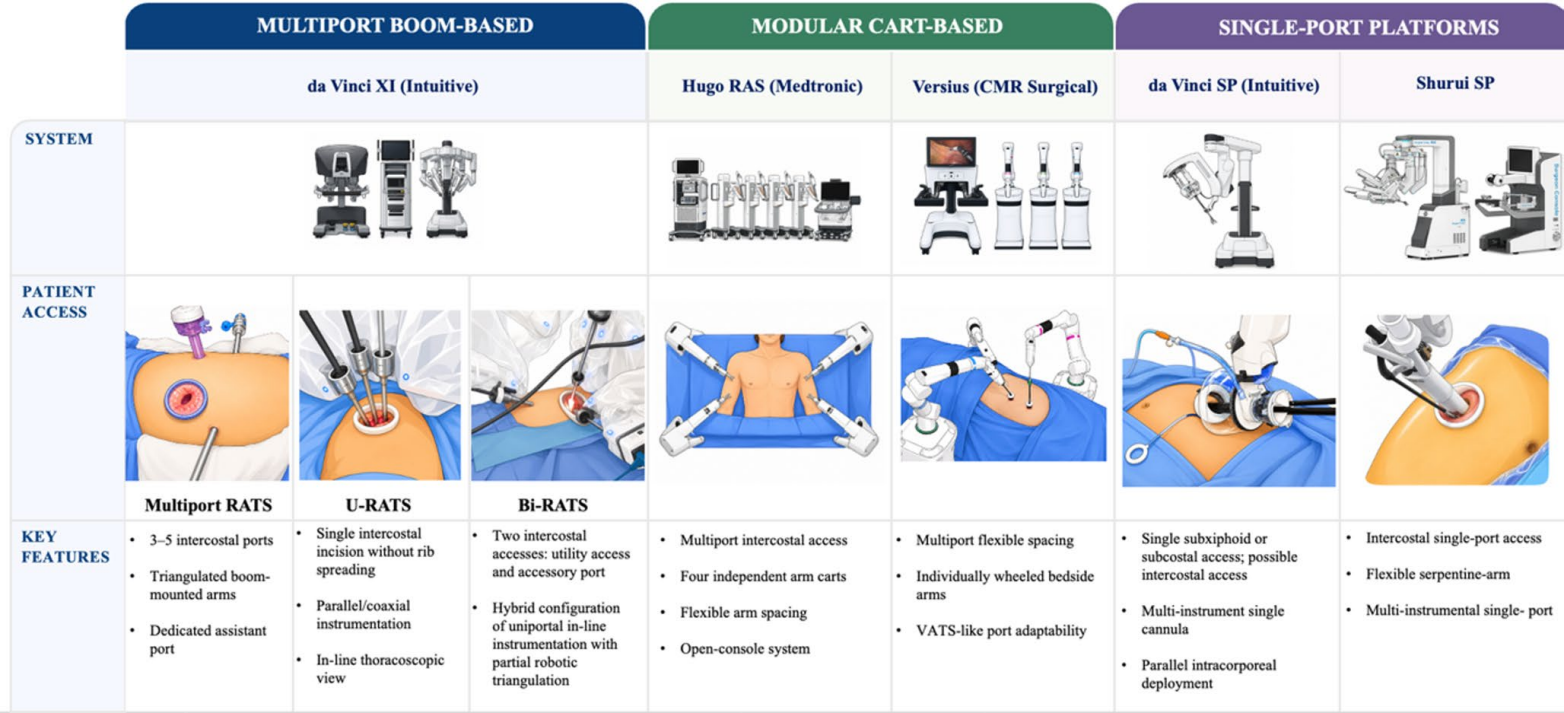
Comparative architecture and access strategies of contemporary robotic thoracic surgery platforms

The robotic surgical landscape is undergoing a fundamental transformation. After more than two decades of near-monopoly by Intuitive Surgical’s da Vinci system, a new generation of platforms, many originating from China, is challenging the status quo through modular architectures, lower acquisition costs, haptic feedback integration, and single-port capabilities [57] (Table 2; Fig. 2). These developments carry profound implications for thoracic surgery, where cost and access barriers have historically restricted robotic adoption to high-volume and resourced centers [58].

**Table 2:** Characteristics of emerging robotic platforms relevant to thoracic surgery platform

Platform	System architecture	Mobility	Single-Port	Access strategy	Force sensing	Thoracic data
**Shurui SP**	Integrated flexible single-port serpentine-arm system	Moderate	Yes	True intercostal single-port access through a single incision	Not reported	Early clinical thoracic series reported
**Toumai** (MicroPort)	Multiport master–slave robotic platform (console, patient cart, vision cart)	Limited	No	Conventional multiport access with separated robotic trocars	Not reported	No Thoracic Data
**EDGE SP1000**	Integrated single-port robotic platform	Moderate	Yes	Single-port access through a dedicated single incision	Not reported	No Thoracic Data
**Hugo RAS** (Medtronic)	Fully modular independent-arm platform	High	No	Multiport intercostal access with independently positioned robotic port	No	Early thoracic feasibility reports
**Versius** (CMR)	Fully modular wheeled-arm system	High	No	Flexible multiport access with customizable port spacing	Yes	Early comparative thoracic studies available

### Chinese and emerging platforms

#### *Shurui SP (Beijing Shurui Technology Co.*,* Ltd.)*

The Shurui SP is China’s first domestically developed single-port robotic system, built around a “deformable dual continuum mechanism”, a fully flexible serpentine-arm design that enables the introduction of multiple articulating instruments through a single intercostal incision [59]. In a prospective multicenter phase 1/2 single-arm registry trial including 35 patients, the system achieved a 100% non-conversion rate, median docking time of 3 min, median blood loss of 10 mL, and 30-day morbidity of 11.4% with no major complications [59]. Compared with the da Vinci SP platform, the Shurui SP incorporates a smaller intercostal-compatible port which allows true intercostal single-port access, whereas the da Vinci SP’s 2.5 cm cannula requires a subcostal incision, limiting its application in complex lung surgeries such as sleeve resection [60]. This design feature may represent a potential technical advantage for selected thoracic procedures, particularly in patients with narrow intercostal spaces. However, no comparative studies have yet demonstrated that this design translates into improved perioperative or oncological outcomes compared with existing robotic platforms.

#### Toumai (MicroPort MedBot).

The Toumai is a multiport master-slave robotic system developed by Shanghai MicroPort MedBot, approved in China for urological surgery in 2022 and undergoing expansion into gastrointestinal and other specialties [61].The Toumai’s primary competitive advantage lies in its substantially lower acquisition cost relative to the da Vinci system, making it a vehicle for expanding robotic access across China and potentially other emerging markets [57]. Thoracic-specific data remain limited, but the platform’s architecture is adaptable to intrathoracic procedures.

#### EDGE SP1000 (Edge Medical).

The EDGE SP1000 is a Chinese-developed single-port robotic platform that has progressed from preclinical porcine validation to early clinical use: Initial clinical experience in gynecologic surgery (single-center feasibility series) reported an average docking time of 5.5 min [62]. No thoracic applications have not been reported to date; however, its compact single-port architecture may be suitable to intercostal access, and the platform’s compact design may facilitate adaptation to thoracic procedures, though this remains speculative pending.

#### Hugo RAS (Medtronic).

The Hugo RAS system, developed by Medtronic, is a modular robotic platform featuring an open surgeon console and four independently positionable robotic arm carts [63]. Each arm is mounted on its own mobile platform, allowing flexible positioning around the patient and adaptability to different operating room configurations. The open console design enhances communication with the operating room team [63]. Clinical validation has been extensive in urology and general surgery. The Hugo system does not currently offer single-port capability or integrated haptic feedback. Its modular arm architecture is particularly relevant for thoracic surgery, where independent arm positioning could facilitate optimal port placement for different thoracic procedures without the geometric constraints of a unified boom.

#### Versius (CMR Surgical).

The Versius system is a UK-developed platform with a fully modular, portable design, in which each robotic arm is a small, individually wheeled unit that can be positioned independently around the operating Table [57]. This architecture enables rapid setup, easy transport between operating rooms, and flexible configurations tailored to specific procedures. The system uses 5 mm wristed instruments and provides force feedback at wrist-level controls [547]. In thoracic surgery, a single-center propensity score-matched study (*n* = 353; 124 RATS vs. 229 uniportal VATS) demonstrated comparable complete resection rates and nodal upstaging, with the robotic group showing a learning curve plateau at approximately 66 cases [64]. Notably, the three lead surgeons had no prior robotic experience, suggesting that the Versius platform may facilitate adoption for VATS-experienced surgeons transitioning to robotics [64]. The system’s portability and lower capital cost compared to da Vinci make it particularly attractive for smaller hospitals and resource-limited settings, though head-to-head comparative data remain limited.

### Thoracic implications: accessibility and competition

The emergence of alternative platforms and non-standard configurations carries implications that extend beyond engineering innovation. The major barrier to widespread of robotic thoracic surgery has been cost, but also maintenance contracts, disposable instrument, and training program expenses [57]. As a result, robotic thoracic surgery in many countries remains confined to a limited number of academic centers, creating significant health inequity [58].

Competition from emerging platforms is expected to reduce costs across the market. Chinese platforms such as the Shurui SP and Toumai are priced substantially below the da Vinci system, and their domestic regulatory approval has facilitated rapid expansion across China’s hospital network [58]. Similarly, the Versius system’s portable, modular design allows a single robotic system to serve multiple operating rooms or even multiple hospitals, potentially improving cost efficiency and reducing per-case expenses [65].

Beyond cost reduction, multi-platform competition may accelerate innovation in areas directly relevant to thoracic surgery, including true intercostal single-port access (Shurui SP), haptic feedback integration (Versius, da Vinci 5), and modular configurations that adapt to the spatial demands of the thorax [9, 59]. The STS has recommended that robotic training curricula become “technology agnostic”, emphasizing transferable principles applicable across different systems and anticipating a multi-platform future [66].

Competition may also reshape surgical training. Currently, robotic training is heavily dependent on industry-sponsored courses tied to a single platform, raising conflict-of-interest concerns [66]. A multi-platform environment could foster independent, society-led training pathways and simulation curricula that are not vendor-specific.

The clinical adaptation phenomenon of U-RATS, Bi-RATS, transcostal SP further underscores how surgical innovation is driven not only by industry but by surgeons who push existing technology beyond its intended boundaries, ultimately influencing the priorities of next-generation platforms.

### Future perspectives in robotic thoracic surgery

The next frontier in robotic thoracic surgery extends beyond hardware refinement and toward the integration of artificial intelligence, augmented reality, fluorescence-guided imaging, autonomous assistance, digital twin technology, and remote surgery. Together, these technologies may transform robotic system into an intelligent surgical partner capable of real-time decision support, predictive modeling, and potentially supervised autonomous task execution [11, 59].

#### Artificial intelligence and computer vision.

AI-driven technologies, including machine learning, deep learning, and computer vision, are advancing across the entire thoracic surgical pathway, from preoperative risk stratification and imaging analysis to intraoperative navigation and postoperative outcome prediction [11]. In thoracic oncology, AI enhances pulmonary nodule detection, histopathological classification, and lymph node metastasis prediction, supporting multidisciplinary decision-making and personalized therapy [11]. Intraoperatively, computer vision algorithms can now identify surgical phases and instruments with accuracy approaching that of expert surgeons [67]. Deep learning models trained on surgical videos can recognize operative steps in real time, enabling automated workflow analysis, performance feedback, and intraoperative decision support [67], although clinical validation remains in early stages.

#### Augmented reality and intraoperative navigation.

Augmented reality (AR) and 3D navigation systems are among the most clinically advanced technologies, with several already in prospective clinical evaluation. These systems overlay preoperative imaging data onto the intraoperative surgical field, providing real-time anatomical guidance during complex resections [68]. A prospective multicenter trial of an AR navigation-guided pulmonary nodule localization system reported a 95.3% localization success rate, with a median localization time of 3.1 min, and no localization-related complications [68, 69].

AR and AI-assisted navigation are particularly relevant for segmentectomy, where accurate identification of bronchovascular anatomy is essential. A prospective multicenter study of a patient-specific virtual navigation system demonstrated near-perfect concordance with operative findings for tumor localization and segment prediction, as well as higher accuracy in predicting resected arteries, veins, and bronchi compared to conventional 2D CT planning, and significantly reduced surgeon workload [70].

The robotic platforms are particularly suited for AR integration. The da Vinci console’s TilePro multi-display allows simultaneous visualization of 3D reconstructions alongside the operative field, creating an immersive experience [70]. Although current evidence is promising, broader clinical adoption will require further standardization and validation in larger prospective studies.

#### Fluorescence guidance.

Indocyanine green (ICG) fluorescence imaging is the most clinically mature technology in robotic thoracic surgery, with applications in intersegmental plane identification, pulmonary nodule localization, and lymphatic mapping [71].

ICG administered intravenously after pulmonary artery ligation delineates the boundary between perfused and non-perfused lung segments under near-infrared imaging [72]. A comparative study demonstrated that the ICG method achieved superior accuracy over the traditional inflation-deflation method even in more complex segmentectomies [71]. However, suboptimal staining can occur in patients with obstructive pulmonary disease or low DLCO values, particularly during lower lobe segmentectomy [73].

ICG has been also used for pulmonary nodule localization in combination with electromagnetic navigational bronchoscopy. Large clinical series of robotic segmentectomies reported a success rate of 86% in correct lesion identification with a median bronchoscopy time of 9 min and R0 resection in all patients [71]. Moreover, a phase II prospective trial showed that near-infrared fluorescence mapping may identify a true intersegmental plane different from the surgeon’s predicted plane in 74% of cases, with a mean additional margin distance of 2.41 cm [73].

A novel approach, ICG inhalation enables tumor margin detection through negative contrast imaging, where the tumor appears dark against fluorescent healthy tissue. This method achieved two-fold higher tumor margin detection efficiency compared to intravenous injection despite using a twenty-fold lower dose of ICG [74].

#### Digital twins.

Digital twin technology, defined as the creation of a dynamic, patient-specific virtual model that mirrors the physical patient in real time, represents a paradigm shift from static preoperative planning to continuous intraoperative guidance [75]. A recent scoping review defined a patient digital twin as a viewable digital replica of a patient, organ, or biological system that contains multidimensional, patient-specific information for decisions support [76].

In thoracic surgery, digital twin applications are emerging in preoperative simulation and intraoperative guidance. Future systems may integrate real-time physiological data, such as ventilation parameters and hemodynamics, with patient-specific anatomical models as a support during surgery while predicting the functional impact of a planned resection in real time [75, 77]. Despite its potential, implementation of this technology faces important challenges including data interoperability, sensor reliability, real-time processing demands, economic costs, and regulatory standards.

#### Autonomous surgical assistance.

Robotic surgery is progressively evolving toward surgeon autonomy, in which the robot performs selected subtasks under surgeon supervision while the surgeon maintains overall procedural control [22]. The most advanced preclinical example is the Smart Tissue Autonomous Robot (STAR), which performed autonomous laparoscopic intestinal anastomosis in a preclinical porcine models (*n* = 4 animals), reporting more consistent needle placement, suture spacing, and suture bite size compared with expert surgeons in that controlled setting [78].

For thoracic surgery, potential autonomous subtasks include automated camera positioning and optimization, tissue retraction, vessel sealing of standardized structures, and stapler deployment along AI-identified intersegmental planes. However, the unique challenges of the thoracic cavity, including respiratory motion, mediastinal shift, and proximity to great vessels, create significant safety requirements that will likely delay autonomous applications [68, 79]. Overall, the path to clinical deployment will require robust safety frameworks, real-time fail-safe mechanisms, and new regulatory paradigms that account for shared human-machine decision-making.

#### Remote surgery and telementoring.

Remote robotic surgery, where the surgeon operates from a console geographically separated from the patient, has become clinically possible through advances in 5G network technology. A major milestone was the first transcontinental single-port robotic thoracic surgery, a right upper lobectomy performed using the Shurui SP platform by a surgical team located more than 8,000 km away, which was completed in 58 min without intraoperative complications [80].

Telementoring represents a more immediately application, where an expert surgeon provides real-time guidance to a remote operating surgeon without directly controlling the instruments. Feasibility studies in robot-assisted minimally invasive esophagectomy (RAMIE) and other procedures have demonstrated reliable communication and high user satisfaction [81].

These technologies could improve the global distribution of thoracic surgical expertise, enabling complex robotic procedures in underserved regions where fellowship-trained thoracic surgeons are unavailable [82]. However, significant barriers remain, such as legal requirements, liability frameworks, cybersecurity concerns, and the need for standardized credentialing across different healthcare systems.

#### Convergence and integration.

The transformative potential of these technologies lies not in any single innovation but in their convergence within a unified surgical ecosystem. Future robotic thoracic operating room may combine AI-driven preoperative planning (3D reconstruction, risk prediction), AR-guided intraoperative navigation (real-time anatomical overlay), ICG fluorescence imaging, digital twin monitoring, computer vision (automated phase recognition and safety alerts), and autonomous assistance for selected subtask execution within a single interconnected robotic platform, potentially integrated with remote mentoring through high-speed networks.

However, the technologies reviewed differ substantially in their level of maturity. Fluorescence-guided imaging has already become an established adjunct in robotic thoracic surgery, while augmented and navigation systems are supported by encouraging prospective clinical studies and are progressively entering clinical practice. By contrast, AI-assisted decision support, digital twins, autonomous surgical assistance, and remote surgery remain largely investigational. Although these technologies have the potential to enhance surgical precision, workflow, and decision-making, robust evidence demonstrating meaningful improvements in patient outcomes, cost-effectiveness, and long-term clinical benefit is currently lacking.

Accordingly, the future impact of robotic thoracic surgery will depend not only on continued technological innovation but also on rigorous clinical validation. Large multicenter prospective studies, standardized evaluation frameworks, robust cybersecurity infrastructure, and appropriate regulatory pathways will be essential to determine which innovations provide genuine clinical value and should be integrated into routine thoracic surgical practice.

## Conclusions

Robotic thoracic surgery has evolved from an emerging technology into a major minimally invasive approach for anatomical lung resection, with expanding applications in increasingly complex procedures. Addressing the central question of this review, the available evidence suggests that the primary demonstrated impact of robotic platform evolutions to date has been on surgeon experience, such as improved ergonomics, enhanced visualization, greater operative autonomy, and expanded procedural capability, rather than on measurable patient outcomes. This does not diminish the value of these advances; improved surgeon ergonomics may reduce fatigue-related errors and facilitate adoption of minimally invasive techniques. Randomized trials have confirmed oncological non-inferiority of RATS compared with VATS, but definitive evidence of patient-level outcomes remains scarce.

Newer innovations as reduced-port approaches and single-port platforms have demonstrated technical feasibility in early-adopter series, but the supporting evidence remains preliminary. More speculative advances, including AI-assisted decision support, augmented reality, and autonomous surgical systems, hold considerable promise but currently lack clinical validation in thoracic surgery.

The emergence of alternative platforms is increasing competition and may accelerate cost reduction, a critical factor for equitable global access. However, technological progress must be accompanied by structured and standardized training pathways and, crucially, by high-quality comparative evidence, including multicenter randomised trials with long-term oncological endpoints.

Ultimately, the future of robotic thoracic surgery will depend not only on continued technological innovation but on the ability to integrate these advances safely, and equitably into clinical practice worldwide and to demonstrate that these translate into meaningful benefit for patients.

## Supplementary Information

Below is the link to the electronic supplementary material.

Supplementary Material 1

## Data Availability

No datasets were generated or analysed during the current study.
