# Supplementary material for: The evolution of robotic-assisted thoracic surgery: current platforms, emerging technologies, and future perspectives - a narrative review
Source: J Robot Surg. 2026 Aug 6;20(1):792. doi: 10.1007/s11701-026-03722-w (PMC13442474; doi:10.1007/s11701-026-03722-w)
Supplement: Supplementary file 1 [file 11701_2026_3722_MOESM1_ESM.docx]

**Supplementary Material**

**Supplementary Methods**

**Structured Literature Search Supporting the Narrative Review**

This review was designed as a **narrative review** supported by a structured literature search to maximize transparency and reproducibility. The search was intended to identify the most relevant literature addressing the historical evolution of robotic-assisted thoracic surgery, current robotic platforms, emerging technologies, surgical applications, training paradigms, and future perspectives. The search was not intended to constitute a formal systematic review.

**Databases**

The following databases were searched:

- PubMed/MEDLINE
- Embase
- Scopus
- Web of Science Core Collection
- Cochrane Library

**Search period:** Database inception through **May 2026**

Language restriction: English

**Search Strategy**

The search strategy combined Medical Subject Headings (MeSH) terms and free-text keywords organized into three conceptual domains:

- Robotic surgery: ("robotic surgical procedures" OR "robot-assisted" OR "robotic-assisted" OR "da Vinci" OR "RATS" OR "Hugo RAS" OR "Versius" OR "Toumai" OR "MicroPort" OR "Shurui" OR "EDGE SP" OR "single-port robotic" OR "telerobotic" OR "remote surgery")
- Thoracic surgery: ("thoracic surgery" OR "thoracoscopy" OR "lobectomy" OR "segmentectomy" OR "thymectomy" OR "esophagectomy" OR "mediastinal surgery" OR "lung resection" OR "VATS" OR "chest wall resection" OR "sleeve resection" OR "lung cancer surgery")
- Domains of interest: ("artificial intelligence" OR "augmented reality" OR "fluorescence" OR "indocyanine green" OR "navigation" OR "digital twin" OR "autonomous" OR "training" OR "curriculum" OR "learning curve" OR "simulation" OR "haptic" OR "cost" OR "outcomes" OR "complications" OR "survival" OR "platform" OR "modular")

Domains 1 and 2 were combined with the Boolean operator AND. Domain 3 was combined with Domains 1 AND 2 using the OR operator to broaden capture while maintaining relevance.

Eligible literature comprised randomized and non-randomized clinical studies, retrospective series, prospective investigations, consensus statements, guidelines, technological reports, and selected preclinical studies relevant to robotic thoracic surgery. Case reports, editorials, letters, and duplicate publications were excluded. Additional studies were identified through manual review of reference lists from relevant articles and previously published reviews (Table 1).

Titles and abstracts were screened for relevance to the objectives of this narrative review. Potentially relevant articles underwent full-text assessment. Additional publications were identified through manual review of reference lists from eligible studies and relevant review articles.

Because the objective of this work was to provide a broad narrative synthesis rather than a formal systematic review or meta-analysis, final article selection was based on scientific relevance, methodological quality, and contribution to the topics addressed.

Given the broad scope of the topic and the heterogeneity of the available evidence, findings were synthesized narratively and organized into thematic sections addressing platform evolution, procedure-specific applications, technological innovations, surgical training, and future directions.

There was no patient or public involvement in the design or conduct of this review.

**Table 1. Search strategy summary**

| Item | Specification |
| --- | --- |
| Date of search | May 2026 |
| Databases and other sources searched | PubMed/MEDLINE, Embase, Scopus, Web of Science, and Cochrane Library |
| Timeframe | Database inception – May 2026 |
| Language | English |
| Inclusion criteria | Randomized trials, prospective and retrospective studies, case series, consensus statements, guidelines, and selected preclinical studies |
| Exclusion criteria | Case reports, editorials, letters, duplicate publications, and non-surgical robotic applications without a thoracic surgical component. |
| Review Type | Narrative Review |
